# A Place in the Brain for Remembering and Forgetting

**DOI:** 10.1371/journal.pbio.0030216

**Published:** 2005-05-10

After the wounded soldier in Dalton Trumbo's *Johnny Got His Gun* slowly realizes he has no limbs, no face, ears, eyes, or mouth—but isn't dead because his mind continues to think—he desperately tries to find a way to reclaim his humanity. He decides to track time: he counts to sixty, files that minute “in one side of his mind … and [begins] counting from one to sixty again.” When he starts to wonder whether he's counting at the right speed, his mind slips “off track” and his figures disappear.

People have been known to exhibit enhanced memory in extraordinary circumstances, but human memory has its limits. Short-term retention of information, called working memory, involves doing at least two things simultaneously: paying attention to the task at hand—in the soldier's case, counting—while processing that information and deciding how to handle it—adding the numbers and keeping a running total. The secondary processing of stored information is referred to as executive control. Many studies suggest that the neural seat of both working memory and executive control—which together encompass planning, creativity, reasoning, abstraction, and most of the other higher-order cognitive properties humans like to claim as their own—lies within the prefrontal cortex. Teasing out the neural components of these overlapping processes has proven challenging.

In a new study, Jonas Rose and Michael Colombo investigate the neural basis of executive control by training homing pigeons to remember or forget a visual stimulus. Recording from the nidopallium caudolaterale (NCL), a region of the avian brain considered analogous to the mammalian prefrontal cortex, the authors show that neurons in the NCL selectively fire when the birds are told to remember and stop firing when they are told to forget.

To test the hypothesis that the NCL plays a role in executive control, the authors trained five pigeons on a directed forgetting test, a variation on the classic match-to-sample test. After viewing sample stimuli consisting of one of two shapes (a circle or dot) or colors (red or white), the birds were cued to remember or forget the sample (signaled by either a high- or low-frequency tone or one of two distinct patterns). A delay period followed these cues. If a forget cue was presented, the trial ended after the delay, and no memory test was given. If the remember cue was presented, the birds were given a memory test in which they saw two stimuli after the delay; if they responded to the sample stimulus (by pecking on a key), they were rewarded with wheat. The pigeons' NCL activity was recorded during the trials.

Eighty-three of the 124 recorded neurons were classified as delay neurons because they showed significantly different activity during the delay period, when memory was required, than during the intervals between trials, when it was not. During the remember trials, neurons showed sustained activation throughout the cue and delay periods; during the forget trials, sustained activation disappeared. To make sure the forget cue was indeed directing the birds to forget the sample stimulus, the authors ran the forget trials again, but this time, tricked the birds and gave them a memory test. The birds consistently performed worse on the forget trials than on the remember trials, confirming the forget cue's effect.

These results suggest that sustained NCL neuronal activation reflects working memory or at least some type of cognitive activity associated with a working memory task. Either way, these findings support the notion that NCL neurons play a role in executive control—what to remember and what to forget—by linking the presence or absence of neuronal activity with remembering and forgetting. And though the avian NCL and mammalian prefrontal cortex clearly differ after 320 million years of divergent evolution, Rose and Colombo make a strong case that they are similar enough to support the NCL's likely contribution to executive control in mammals as well. And they suggest that seeing such similarities between the bird and human brain forces us to reexamine not only our notions of how these structures operate but also our hubris in thinking our biology and nature is unique.[Fig pbio-0030216-g001]

**Figure pbio-0030216-g001:**
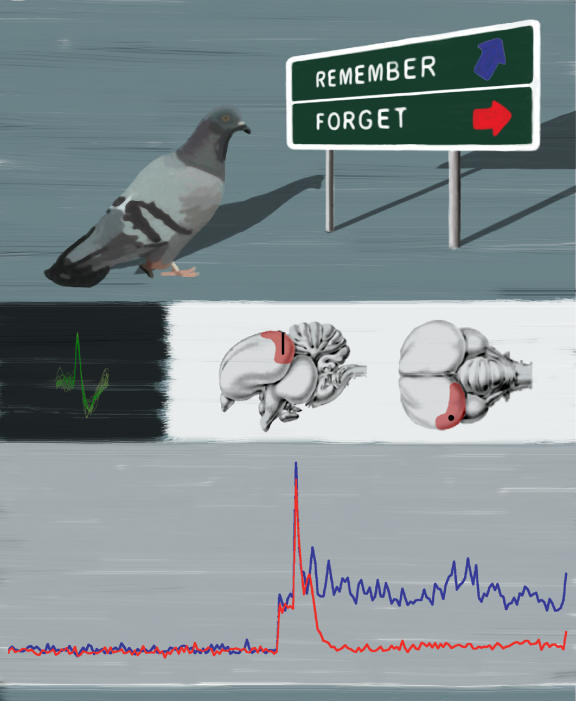
Neurons in the avian version of the prefrontal cortex fire when pigeons perform memory tasks and may play a role in higher cognitive processes such as decision making and reasoning

